# SGLT2 inhibitors as add on therapy in type 2 diabetes: a real world study

**DOI:** 10.1186/s40200-017-0308-4

**Published:** 2017-06-30

**Authors:** Héctor Eloy Tamez-Perez, Enrique Delgadillo-Esteban, David Soni-Duque, Mayra Ivonne Hernández-Coria, Alejandra Lorena Tamez-Peña

**Affiliations:** 1 Subdirección de Investigación, Facultad de Medicina. Universidad Autónoma de Nuevo León, Dr. Aguirre Pequeño, Mitras Centro, C.P. 64460 Monterrey, Nuevo León Mexico; 2 División de Medicina Interna “Modelo Nova”, Clínica Nova, Monterrey, Nuevo León Mexico

**Keywords:** Dapagliflozin, Canagliflozin, Diabetes Mellitus, Type 2, Effectiveness research, comparative, Hemoglobin A, Glycosylated

## Abstract

**Background:**

Type 2 diabetes mellitus (T2DM) is a progressive chronic disease associated with severe microvascular and macrovascular complications.

Our aim is to assess the real world effectiveness of SGT" inhibitors in achieving metabolic therapeutic goals.

**Methods:**

A retrospective, observational study. Inclusion criteria for patients were a previous diagnosis of type 2 diabetes mellitus, age > 18 years, patients receiving either dapagliflozin 10 mg and/or canagliflozin 300 mg. We excluded pregnant patients, patients with type 1 diabetes mellitus and acute metabolic complications of diabetes. Patients included in the analysis were enrolled in a health plan at least 6 months prior to the index date (baseline period) and in the 6 months following the index date (follow-up period). Achievement of glycated hemoglobin goals were established as <7%.

**Results:**

We screened 2870 Mexican patients; 288 (10.03% received SGLT2 inhibitors). Mean age for both groups of patients was 57.68 ± 11.06 years. The dapagliflozin control rate was 19.56% and the canagliflozin control rate 18.96%. Monotherapy with SGLT2 inhibitors was used in 21 patients (6.25%). Overall HbA1c goals were met in 56 patients (19.44%) with similar results with dapagliflozin or canagliflozin. The combination of SGLT2 inhibitors and sulfonylureas had the highest control rate (30.30%) compared to other regimens. Monotherapy was present in 6.25%. Insulin requirement was associated with poor control (2.8% vs. 18.05%, *P* < 0.05, 95% CI [0.07, 0.84]). Combination therapy with DPP4 inhibitors was associated with better control (*P* < 0.05, 95% CI, [1.10, 3.92]).

**Conclusion:**

No difference between the drugs was observed. Real-world effectiveness data of SGLT2 inhibitors show that the percentage of patients reaching metabolic goals is low. SLGT2 inhibitors were used more frequently as combined therapy.

## Background

Type 2 diabetes mellitus (T2DM) is a progressive chronic disease characterized by insulin resistance and a progressive insulin secretory defect associated with severe microvascular and macrovascular complications [1]. Until recently there was little evidence for cardiovascular risk reduction with glucose-lowering therapies in type 2 diabetes (T2DM). However, within the last year the results of two trials (EMPA-REG and LEADER) have demonstrated substantial cardiovascular benefit with two agents: SGLT2 inhibitor empagliflozin and glucagon-like peptide-1 (GLP-1) analogue liraglutide. Many large randomized control trials have demonstrated a significant reduction in microvascular events in patients treated with hypoglycemic agents leading to a reduced HbA1C [2].

The progressive nature of the disease means that most patients require increasingly intensive pharmacologic interventions often with multiple classes of diabetes medications. Given this issue, the American Diabetes Association (ADA) guidelines recommend HbA1C therapeutic goals <7% [3] to reduce morbidity and complications in T2DM.

A class of oral antihyperglycemic agents (AHA) that has the potential to address these needs is now available. Sodium glucose co-transporter type 2 (SLGT2) inhibitors are becoming a promising therapy for T2DM. These drugs reduce hyperglycemia by blocking renal glucose reabsorption, in the proximal tubule of the kidney. This induces glucosuria which lowers serum glucose and also induces diuresis [3].

A systematic review evaluating the effectiveness of SGLT2 has shown that HbA1C is decreased in a range of 0.32% to 1.17% [4]. Other effects reported are a slight reduction of triglycerides [5], blood pressure, and weight, and a low potential for hypoglycemia [6, 7]. Recently, the EMPA- REG study showed that patients, with a high-risk for CVD, receiving empagliflozin had a lower rate of deaths from CVD [6].

However, few studies [2, 4, 5] have been conducted evaluating the effectiveness of SGLT2 inhibitors, either combined with another AHA or as monotherapy, for reaching ADA HbA1C therapeutic goals in a real-world setting.

The objective of this study is to assess the effectiveness of SLGT2 inhibitors (dapagliflozin, canagliflozin) in achieving HbA1C goals in patients with T2DM in a private Mexican outpatient clinic.

## Methods

This cohort study used patient data obtained from a large health plan database from an outpatient clinic (Hospital Clinica Nova, San Nicolas de los Garza, Nuevo Leon, Mexico) during 2014–2016. Inclusion criteria were a diagnosis of T2DM, age > 18 years, and receiving either dapagliflozin 10 mg (Forxiga®, AstraZeneca) or canagliflozin 300 mg (Invokana®, Janssen) with or without background metformin therapy and not achieving glycemic targets. We excluded pregnant patients, patients with type 1 diabetes mellitus, and patients with acute metabolic complications (diabetic ketoacidosis, hyperglycemic hyperosmolar state) and those with poor treatment adherence from primary analysis, the other inhibitor drug class (empagliflozin) it’s currently not available in our clinic. Baseline characteristics (age, gender, comorbidity, drug use, and insulin requirements) were reported. Achievement of glycated hemoglobin goals was established as <7% following ADA 2016 recommendations [3]. Patients included in the analysis were enrolled in the health plan for at least 6 months prior to the index date (baseline period) and 6 months following the index date (follow-up period). The study was approved by the local ethics committee. Statistics were reported as frequencies, percentages, central tendency, and dispersion measures. For continuous variables, normality testing was done using the Shapiro-Wilk test. For testing difference in means, a two sample t-test or Mann Whitney U test was used. For discrete variables, Pearson χ2 or the Fisher exact test was used as needed. A *P* value <0.05 was considered statistically significant. Statistical analysis was made using R software v 3.3.1 [7].

## Results

We screened 2870 patients; of these 288 (10.03%) received SGLT2 inhibitor therapy. Monotherapy with SGLT2 inhibitors was present in 21 (6.25%). Metformin was the most frequent AHA (57.29%) combined with SGLT2 inhibitors. Most patients used at least 2 additional different antidiabetic drugs (46.87%). Co-morbidities were present in 38 (13.19%) patients. Fifty-three (18.40%) patients required insulin.

In the dapagliflozin group, mean patient age was 57.68 ± 11.06 years (range 34 to 82). According to gender, there were 135 (58.69%) women and 95 (41.3%) men. HbA1C goals were met in 19.56% of patients. In the canagliflozin group, 58 cases were included. Mean patient age was 58 ± 10.56 years (range 30 to 83). According to gender, 28 (50%) patients were men and 28 (50%) women. HbA1C goals were met in 18.96% of patients. The canagliflozin group had an average reduction on the HbAC1 levels from 9.72% to 8.69% and the dapagliflozin group from 9.90% to 8.83%.Both therapies had similar reductions of HbA1C with no significant difference between them. Overall HbA1C goals were met in 56 (19.44%) patients. The combination of SGLT2 inhibitors with sulfonylureas, and dipeptidyl peptidase 4 (DPP4) inhibitors had the highest control rate (30.30%/26.55%) compared with other regimens. Mild weight loss was reported (3.2 vs. 2.7 kg, *P* = 0.657) with no significant difference between dapagliflozin and canagliflozin.

Patients with insulin requirement had poorer control (7.75%) than non-insulin users (22.13%, *P* < 0.05, 95% CI [0.07, 0.84]). No severe hypoglycemic events, ketoacidosis, and severe urinary tract infections were reported. However, a higher frequency of urinary tract infections (OR 2.3, 95% CI [1.81, 2.78]) and mycotic infections (OR 4.02, 95% CI [4.02, 5.18]) in women, (OR 3.17 95% CI [2.63, 3.71]) and men in the treated group was reported. Blood pressure and lipid profiles were not included in the analysis due to incomplete data. All results are summarized in Table 1.

**Table 1:** ADA Hb1Ac goals in patients with type 2 diabetes

Variable	HbA1c < 7	HbA1c > 7	p	95% CI	OR
n	56 (19.44)	232 (80.55)	–	–	
Age (years)	56.89 ± 10.62	57.99 ± 10.98	0.4938^c^	[-2.07,4.26]^a^	–
Female (%)	29 (17.68)	135 (82.32)	0.472564^d^	[0.69,2.43]	1.3
Male (%)	27 (21.77)	97 (78.23)			
Insulin Requirement (%)					
No	52 (22.13)	183 (77.87)			
Yes	4 (7.55)	49 (92.45)	<0.05^a, e^	[0.07,0.84]	0.29
Combination with other ant diabetics (%)					
Saxaglyptin	29 (26.85)	79 (73.15)	<0.05^a, d^	[1.1,3.92]	2.08
Sulfonylureas	10 (30.3)	23 (69.7)	0.1495^d^	[0.78,4.67]	1.98
Metformin	38 (23.03)	127 (76.97)	0.103^d^	[0.91,3.44]	1.75
Pioglitazone	8 (23.53)	26 (76.47)	0.6817^d^	[0.49,3.24]	1.32
Acarbose	6 (21.43)	22 (78.57)	0.9777^d^	[0.36,3.11]	1.15
Monotherapy	21 (20.39)	82 (79.61)	0.8834^d^	[0.57,2.08]	1.1
Sitagliptine	18 ( 12.59 )	125 (87.41)	<0.01^b,d^	[0.21,0.78]	0.41
Drug use (%)					
Statin	30 (18.75)	130 (81.25)	0.854714^d^	[0.48,1.7]	0.91
Aspirin	20 (15.5)	109 (84.5)	0.16998 d	[0.32,1.19]	0.63
Comorbidity (%)					
Dyslipidemia	6 (17.65)	28 (82.35)	0.959111^d^	[0.28,2.31]	0.87
Neuropathy	0 ( 0 )	3 (100)	0.902737^e^	[0,10.1]	–
Nephropathy	0 ( 0 )	1 (100)	1^e^	[0,161.17]	

*95% CI* 95% confidence interval; *OR*odds ratio

^a^P < 0.05

^b^P < 0.01

^c^two-sample t-test

^d^Pearson χ2

^e^Fisher´s exact test

## Discussion

In our clinic, the use of SGLT2 doesn’t correspond to a first line treatment. Despite the decrease in HbA1C, few patients achieved ADA glycated hemoglobin goals.

Second-line pharmacotherapy selection for patients with T2DM is debated. Experts recommended patient- specific considerations. SGLT2 are a promising new class of diabetes pharmacotherapy that particularly shows favorable effects on weight, low potential of hypoglycemia and a reduction of cardiovascular risk [2, 4, 5].

We observed a significant reduction of HbA1C, similar to a trend in the use of SGLT2 inhibitors as combination therapy compared with previous studies, lowering HbA1C by 1%–1.5%. The majority of clinical trials evaluate moderately uncontrolled diabetes and there is limited data available describing the efficacy of these drugs in patients with poor metabolic control; however, the reduction in glucotoxicity with SGLT2 and improvement insulin sensitivity may result in an increase benefit in patients with a higher HbA1C [4, 5]. We noted a steady increase in control rates in patients receiving SGLT2 inhibitors in addition to sulfonylureas and DPP4 inhibitors, but our results are less than those of controlled clinical trials, attributable to not following current guidelines that recommend stepwise intensification. Rosentock et al. reported a better response in HbA1C goals with triple therapy saxagliptin, metformin and dapagliflozin (41%) and the dual addition of saxagliptin and dapagliflozin (22%). They reported a reduction of HbA1C levels of 1.47% [8]. This HbA1C reduction in the dual add-on group was less than the summation of the reductions seen in the monotherapy arms. No significant differences in control between canagliflozin and dapagliflozin were noted, probably due to class effect. In a recent study, empagliflozin has been noted to reduce CVD risk [6]; however, it was not evaluated here due to unavailability. Metformin is recommended as first- line treatment for patients with T2DM due to its well-established efficacy, safety, low cost, and data demonstrating a reduction in risk of cardiovascular events. SGLT2 as monotherapy is an option in patients with contraindications to the use of metformin or who do not tolerate metformin; in our group alone, this was considered in only 21 patients (6.25%).

Merocvi and cols reported that SGLT2 inhibitors as monotherapy improve insulin sensitivity and reduce the progressive loss of beta-cell function and insulin resistance; unfortunately, limited long- term data are available [9].

Weight loss is a desirable outcome in most patients with T2DM due to the positive impact of glycemic control, insulin sensitivity, and comorbidity. Our study results do not differ importantly from other publications [3, 4]. Weight loss appears to be sustained for up to 102 weeks [4, 6].

The positive effects on cardiovascular outcomes are highly promising. Long term data, including primary cardiovascular prevention populations and utilizing other SGLT2, will be needed to fully assess the cardiovascular outcomes with this class of medications.

SGLT2 inhibitors have been associated with an increased risk of genitourinary infections and polyuria due to increased glucose concentrations in the urine [10–12]. Our rates appear modestly increased and no severe adverse effects were reported. A recent study comparing trial against real world data concluded that extrapolation of the trials and the results in the real world do not match [2]. Further research is needed to clarify and validate the benefits of SGLT2 at the translational biology level as well as its impact on clinical medicine [13].

We must become familiar with autonomous medical education and perform precision medicine. In diabetes, the selection of treatment should be patient specific and incorporate knowledge of potential adverse effects, the required HbA1C reduction to archive the goal, cost, weight consideration, co-morbid medication conditions, and patient preferences.

Given the cost of this group class therapy, it should be stopped in patients that fail to respond.

An important strength of the study is the comparison of the overall effectiveness of SGLT2 inhibitors alone or combined in a private clinic setting where all the physicians are internal medicine specialists in a real-world setting. Also, the study evaluated the impact of multiple combined regimens of SGLT2 inhibitors on glycemic goals. The limitations of the study include that data was retrieved from electronic medical records that contain limited data for important measurements such as blood pressure or lipid profile of a small sample of patients this has been analyzed recently and we accept that there is a need for real life pragmatic trials; the limitations are problematic [14]. Differences in the size of population analyzed in the dapagliflozin and canagliflozin groups appear due to when each drug was introduced to the clinic and which one it’s prescribe by each physician. Empagliflozin was excluded from the analysis because it’s fairly new option in our clinic.

Also, patients were treated in a secondary-level outpatient clinic. HbA1c measurements were made only during a 6-month period, and the study assessed control outcomes without reporting adjustments of important comorbidities such as chronic kidney disease, estimated glomerular filtration rate reduction may negatively affect the efficacy of SGLT2 inhibitors [15].

## Conclusions

The results were similar for both drugs. Our results are not comparable to clinical trials, which should be performed carefully in the real-world and the effectiveness data of SGLT2 inhibitors show that the percentage of patients reaching metabolic goals is low. SLGT2 inhibitors were used more frequently as combined therapy. Further research is needed to validate these findings in other T2DM patient populations.

## Abbreviations

ADA: American Diabetes Association
AHA: Oral antihyperglycemic agents
CHD: Risk of coronary heart disease
CVD: Cardiovascular disease
DPP4: Dipeptidyl peptidase 4
HbA1C: Glycated hemoglobin
SGLT2: Sodium glucose co-transporter type 2
T2DM: Type 2 diabetes mellitus
